# Seasonal variation in the onset of acute calcific tendinitis of rotator cuff

**DOI:** 10.1186/s12891-020-03773-6

**Published:** 2020-11-12

**Authors:** Ryogo Furuhata, Noboru Matsumura, Akira Yoshiyama, Yusaku Kamata, Masaaki Takahashi, Hideo Morioka

**Affiliations:** 1 Department of Orthopaedic Surgery, National Hospital Organization Tokyo Medical Center, 2-5-1, Higashigaoka, Meguro-ku, Tokyo, 152-8902 Japan; 2 Department of Orthopaedic Surgery, Keio University School of Medicine, Shinjuku-ku, Tokyo, Japan

**Keywords:** Calcific tendinitis, Rotator cuff, Resorption, Season, Shoulder

## Abstract

**Background:**

Calcific tendinitis of the rotator cuff is a disorder that causes acute onset of shoulder pain when spontaneous resorption of the calcification occurs. However, factors that trigger calcium resorption have not been clarified. The present study aimed to investigate the association between the onset of calcium resorption in calcific tendinitis and the season of onset.

**Methods:**

We retrospectively reviewed 195 patients (female, 116; male, 79; mean age, 62.6 ± 14.2 years; median age, 62 [52, 73] years) diagnosed with the postcalcification stage of calcific tendinitis, which was defined as acute calcific tendinitis in this study, between 2006 and 2018. The onset date of acute calcific tendinitis for each patient was collected from clinical notes. We evaluated the incidence of acute calcific tendinitis in each season and month. Furthermore, we investigated the correlation between the incidence of acute calcific tendinitis and the mean monthly temperature or humidity levels for each year.

**Results:**

The most common season of acute calcific tendinitis onset was summer (35.4%), followed by spring (24.6%), autumn (24.1%), and winter (15.9%) (*P* = 0.002). Monthly analyses showed the highest peak of onset was in July (15.4%) and the lowest peak was in February (3.1%) (*P* = 0.022). The incidence of acute calcific tendinitis had a weak association with mean monthly temperature (R^2^ = 0.066; *P* = 0.001) but was not associated with mean monthly humidity levels (R^2^ = 0.018; *P* = 0.099).

**Conclusions:**

This study provides new information on seasonal variation of acute calcific tendinitis onset. The results of this study indicated that the onset of calcium resorption occurs most frequently in the summer in Japan; however, the reasons for seasonal variation remain unclear, and further studies will be needed.

**Level of evidence:**

Level III.

**Supplementary Information:**

The online version contains supplementary material available at 10.1186/s12891-020-03773-6.

## Background

Calcific tendinitis of the rotator cuff is a common disorder, in which calcified deposits are formed in the rotator cuff, followed by spontaneous phagocytic resorption [1, 2]. Uhthoff and Loehr [2] proposed that the evolution of calcific tendinitis can be divided into three distinct stages: precalcification, calcification, postcalcification. The calcification stage of calcific tendinitis is classified into formative phase, resting phase and resorptive phases [2]. Although the formative phase of calcific tendinitis is characterized by mild chronic pain or no symptoms, when spontaneous resorption of the calcification occurs, patients present with acute onset of severe shoulder pain, making shoulder joint motion difficult [1]. With resorption of calcium, patients enter the postcalcification stage, in which the tendon heals with fiber realignment and resolution of calcium deposits [2]. Thus, it is important to identify factors that induce calcium resorption in the rotator cuff. However, the etiology of calcific tendinitis remains unclear, and factors triggering calcium resorption have not been fully understood. In addition, the seasonality of calcific tendinitis has not been reported.

The present study aimed to investigate the association between the onset of calcium resorption in calcific tendinitis and the season of onset.

## Methods

This study was approved by our hospital’s Independent Ethics Committee (No. R19–135).

### Patient selection

**Fig. 1:**
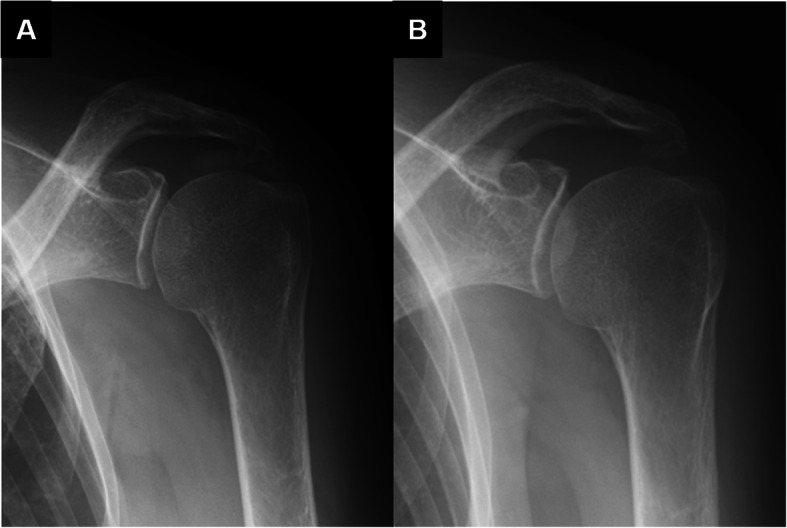
Plain radiographs of acute calcific tendinitis of the rotator cuff. (**a**) Plain radiography performed at the onset of acute calcific tendinitis shows calcium deposition in the rotator cuff in an 80-year-old woman. (**b**) After 3 weeks from the onset, calcium deposition has disappeared

We performed a retrospective study that reviewed patients who were diagnosed with calcific tendinitis at one general hospital in Tokyo between January 2006 and December 2018. Our institution accepted outpatient visits without a referral letter or reservation during the same period. The diagnosis of calcific tendinitis was made from physical findings and plain radiograph images. In the resorptive phase of calcific tendinitis, symptoms of pain usually disappeared 1–2 weeks after the onset [2]. Therefore, our study included patients with limited active motion due to intense acute pain with a well-defined onset date within 2 weeks, in addition to the presence of calcific deposition in the rotator cuff on plain radiographs (Fig. 1). This condition was defined as acute calcific tendinitis in this study. In conventional calcific tendinitis staging, this is equivalent to the postcalcification stage after the resorption of the calcium [2]. Conversely, even if patients present with calcific deposition on plain radiographs, those who experienced chronic pain or those predominantly with pain arc signs were considered to have symptoms during the calcium formative phase and were excluded. In addition, because pain medications, such as non-steroidal anti-inflammatory drugs (NSAIDs) or acetaminophen, taken before or during the clinical visit, might have affected the onset of acute calcific tendinitis, patients with a history of these medications within 1 month before the onset of pain were excluded from this study.

We identified 257 patients who met the inclusion criteria. 57 patients were excluded from this study because they were considered to be suffering from the symptoms during the calcium formative phase. In addition, 5 patients were excluded due to history of pain medications for lumbar spinal canal stenosis or post-surgical pain management within 1 month before the onset of pain. Among the remaining 195 patients (mean age, 62.6 ± 14.2 years; median age, 62 [52, 73] years; range, 27–104 years), 116 (59.5%) were female. The affected shoulder was the right in 118 patients (60.5%) and the left in 77 patients (39.5%). In this study, no patient developed acute calcific tendinitis on both sides during the study period. Calcific tendinitis has been reported to be associated with diabetes and thyroid disorders [3–6], and in this study, 21 patients (10.7%) had a history of diabetes, and 4 patients (2.1%) had a history of thyroid disorders. In terms of level of activity, 189 patients (96.9%) were independent in daily life, 5 patients (2.6%) were being hospitalized for chemotherapy or radiotherapy for carcinoma, and 1 patient (0.5%) needed wheelchair assistance. Two patients (1.0%) were athletes. Eight patients (4.1%) developed acute shoulder pain due to trauma, 18 patients (9.2%) due to heavy work, and 8 patients (4.1%) due to sports. However, the cause of shoulder pain was unknown in the remaining 161 patients (82.6%).

In addition, in this study, 155 patients were able to follow-up on the symptoms. Among these 155 patients, 148 (95.5%) achieved complete restitution through conservative treatment (subacromial bursa injections in 11 patients; pain medications, such as NSAIDs and acetaminophen, in 55 patients; both injections and pain medications in 76 patients; and only follow-up in 6 patients). However, 7 patients (4.5%) had residual pain 6 months after the onset of pain.

### Outcome measures

A single examiner obtained the onset date of acute calcific tendinitis from clinical notes. The date of each event was categorized into 4 seasons and 12 months. The seasons were categorized as every 3 months (the period from March to May was defined as spring, that from June to August was summer, that from September to November was autumn, and that from December to February was winter). The incidence of acute calcific tendinitis in each season and month was tabulated and comparatively evaluated.

We also collected the local climate data from the Japan Meteorological Agency and evaluated the association between the incidence of acute calcific tendinitis and mean monthly temperature or mean monthly humidity levels for each year. Mean monthly temperature and mean monthly humidity levels over the entire period of 13 years between 2006 and 2018 in Tokyo are briefly summarized in Table 1.

**Table 1:** Mean monthly temperature and mean monthly humidity levels between 2006 and 2018 in Tokyo

Month	Mean monthly temperature (°C)	Mean monthly humidity levels (%)
January	5.9 ± 0.8	46.7 ± 5.3
February	6.5 ± 0.9	52.3 ± 4.2
March	10.0 ± 1.1	54.5 ± 6.3
April	14.7 ± 1.1	60.4 ± 5.8
May	19.7 ± 0.7	64.2 ± 3.8
June	22.5 ± 0.7	71.8 ± 4.5
July	26.6 ± 1.0	74.2 ± 3.7
August	27.8 ± 1.1	72.7 ± 4.9
September	24.1 ± 1.1	72.9 ± 6.9
October	19.0 ± 0.7	68.2 ± 5.4
November	13.4 ± 0.9	62.3 ± 6.5
December	8.5 ± 1.1	53.8 ± 3.5

In addition, to investigate whether the season of pain onset or age affects the relief of the symptoms of acute calcific tendinitis, we divided the 155 patients who completed the follow-up of the symptoms into the complete restitution group and the incomplete restitution group, in which patients had residual pain 6 months after pain onset. We then compared the frequency of incomplete restitution between seasons, as well as the mean age between the complete restitution group and the incomplete restitution group.

### Statistical analysis

All statistical analyses were conducted using the SPSS software program (Version 26.0, IBM Corp., Armonk, NY, USA). Goodness-of-fit tests were performed for season and month of onset of acute calcific tendinitis. Simple linear regression was used to test for the correlation between the incidence of acute calcific tendinitis and the mean monthly temperature or humidity levels. We used Student’s *t*-tests to compare the average of continuous values (age between restitution group and incomplete restitution group) and chi-square tests to compare the proportion of discrete variables (the frequency of incomplete restitution among seasons). The threshold for significance was set at *P* < 0.05.

## Results

The most common season of acute calcific tendinitis onset was summer (35.4%), followed by spring (24.6%), autumn (24.1%), and winter (15.9%) (*P* = 0.002). These distributions were observed in both female and male patients in the analysis with respect to sex; however, significant differences were only observed in female patients (*P* = 0.009) (Table 2). Monthly analyses showed that the highest peak was in July (15.4%) and the lowest in February (3.1%) (*P* = 0.022) (Table 3). There was a weak association between mean monthly temperature and the incidence of acute calcific tendinitis (R^2^ = 0.066; *P* = 0.001), but no significant association was found between mean monthly humidity levels and the incidence of acute calcific tendinitis (R^2^ = 0.018; *P* = 0.099).

**Table 2:** Seasonal distribution of the onset of acute calcific tendinitis

	Number of patients	Winter n (%)	Spring n (%)	Summer n (%)	Autumn n (%)	Goodness of fit	
						χ^2^	P
**Total**	195	31 (15.9)	48 (24.6)	69 (35.4)	47 (24.1)	14.9	0.002
**Females**	116	19 (16.4)	27 (23.3)	44 (37.9)	26 (22.4)	11.7	0.009
**Males**	79	12 (15.2)	21 (26.6)	25 (31.6)	21 (26.6)	4.6	0.204

*n*; number

**Table 3:** Monthly distribution of the onset of acute calcific tendinitis

Month	Number (%) out of 195
January	13 (6.7%)
February	6 (3.1%)
March	16 (8.2%)
April	18 (9.2%)
May	14 (7.2%)
June	20 (10.3%)
July	30 (15.4%)
August	19 (9.7%)
September	15 (7.7%)
October	18 (9.2%)
November	14 (7.2%)
December	12 (6.2%)

*P* = 0.022, by goodness-of-fit test

In addition, there were no significant differences in the frequency of patients who experienced incomplete restitution between each season of pain onset (Supplementary Table 1). However, the mean age of patients who went through incomplete restitution was significantly younger than that of patients who achieved complete restitution (*P* = 0.032) (Supplementary Table 2).

## Discussion

In this study, we investigated the seasonality of acute calcific tendinitis onset in Japan. The results of our study indicated that the occurrence of acute calcific tendinitis exhibited a seasonal distribution characterized by a summer peak in July. Additionally, this study suggested that the mean monthly temperature can affect the onset of acute calcific tendinitis.

To date, seasonal variation of calcium resorption in calcific tendinitis has not been clarified. Regarding musculoskeletal pain, some previous studies have reported that cold or humid weather conditions negatively influence the symptoms of patients with chronic pain [7, 8]. However, our results do not agree with those of previous reports on musculoskeletal chronic pain. This discrepancy suggests that the mechanism of pain associated with the onset of calcium resorption differs from that of musculoskeletal pain. Conversely, acute gout attack, a type of microcrystalline arthritis, has been reported to exhibit seasonality, with its onset being less frequent in the winter and more frequent in the spring to summer seasons [9–14]. These results are similar to those of seasonal distributions in the present study. However, the incidence of acute gout attack has been reported to have no significant association with mean monthly temperature [14]. Thus, changes in physical activity, serum uric acid/lipid/cortisol levels, diet, and alcohol consumption associated with seasons were suggested as triggering factors of acute gout attack [11, 14, 15].

Based on the results of this study, the mechanism behind seasonal variation of acute calcific tendinitis is unclear; however, several possible hypotheses can be raised. One hypothesis involves the regulation of phagocytic activity of immune cells by body temperature. Pathological findings in the resorptive phase of calcific tendinitis include macrophage and multi-nucleated giant cells surrounding broken-up calcium deposits, and phagocytosis of the calcification by these cells is suspected to cause the calcium resorption [6, 13, 16]. Furthermore, diffusion of apatite crystals from calcification into the subacromial bursa causes acute severe pain [17]. While the function of macrophages can be regulated by various factors, transient receptor potential melastatin 2 (TRPM2), a thermosensitive channel expressed in a wide range of immunocytes including macrophages, has also been identified to regulate macrophages [18]. TRPM2 contributes to enhancing the phagocytic activity of macrophages when body temperature is elevated [18]. When the ambient temperature is elevated in the summer, the opportunity of increasing skin temperature also increases, which may predispose the activation of phagocytosis in macrophages. This mechanism could possibly explain the predominance of the onset of acute calcific tendinitis in the summer. Another hypothesis is that repetitive microtauma owing to increased physical activity in the summer may affect the immune responses in the rotator cuff. In this study, 17.4% of patients developed the symptoms due to trauma, heavy work, or sports. This finding supports the possibility that increased physical activities may also affect the onset of acute calcific tendinitis. However, further studies are needed to clarify the mechanism of the seasonal variation of acute resorption of calcific tendinitis.

In contrast, the results of our study did not suggest a correlation between the season of pain onset and the disappearance of symptoms. One previous study reported that 90% of symptomatic calcific tendinitis of the rotator cuff is relieved by conservative treatment [19], and in this study, similar results were obtained, where the symptoms disappeared in 95.5% of patients by conservative treatment. In a previous study, multivariate analysis identified female gender as a significant risk factor against conservative treatment [20]; however, the prognostic factors of treatments for acute calcific tendinitis remain largely unknown. The results of our study suggested that the season of pain onset may not have affected the symptom course, but younger age may be a negative prognostic factor of conservative treatment.

In addition, the results of our study also suggested that the epidemiology of the calcific tendinitis in resorptive phase does not necessarily match the epidemiology of calcific tendinitis overall. In a previous study that investigated 6061 volunteers [21], calcific deposits in the rotator cuff were observed in 2.7% of them, but among those with calcific deposits, 34.6% were symptomatic, and in particular, only 8.9% exhibited acute shoulder pain. In this study, only patients in the calcium resorptive phase exhibiting acute shoulder pain were included. Thus, even though calcific tendinitis is a common disease, the number of subjects who developed this disease in this study was smaller for the study period of 13 years. Calcific tendinitis has been reported to be associated with diabetes and thyroid disorders [3–6]. However, in this study, patients with a history of diabetes and thyroid disorders were 10.7 and 2.1%, respectively, and these were almost the same as the incidence of diabetes (11.2% [22]) and thyroid disorders (up to 10% [23]) among the Japanese, suggesting no correlation between the onset of calcific resorption in calcific tendinitis and history of these diseases. In addition, 21.3–46.4% of patients were reported to exhibit bilateral calcific deposits [20, 21], but in this study, no patient developed bilateral calcific resorption during the study period. In terms of age, calcific tendinitis of the rotator cuff occurred commonly in the patients aged 30 to 50 years [21], but the mean age of patients in this study was slightly older, at 62.8 ± 14.2 years, suggesting that calcific resorption may have developed after a certain period of time after the formation of calcium. These findings suggest that the epidemiology of calcific tendinitis in the resorptive phase is not representative of the epidemiology of calcific tendinitis overall.

This study has several limitations. First, because this is an observational study, it can be influenced by residual confounding owing to bias caused by factors not measured in this study. For example, the concurrence of the rotator cuff and adhesive capsulitis can cause to trigger the onset of acute calcific tendinitis, but these factors were not considered in this study. Second, as this study was conducted in a general and emergency hospital in Tokyo, there were more patients in need of surgery and more acute diseases compared to medical practitioners, which might have resulted in selection biases and a smaller sample population. In addition, the ethnicity was limited to the Japanese population. Thus, the present results may not represent the general population.

## Conclusions

This study provides new information on seasonal variation of acute calcific tendinitis onset. The results of this study indicated that the onset of calcium resorption occurs most frequently in the summer in Japan; however, the reasons for seasonal variation remain unclear, and further studies will be needed.

## Supplementary Information

**Additional file 1: Supplementary Table 1.** Association between the restitution and season of the pain onset of acute calcific tendinitis.

**Additional file 2: Supplementary Table 2.** Association between the restitution and age of patients in acute calcific tendinitis.

## Data Availability

Data that support the findings of this study are available from the corresponding author on reasonable request.

## Abbreviations

NSAIDs: non-steroidal anti-inflammatory drugs
TRPM2: transient receptor potential melastatin 2
