# Supplementary material for: Seasonal variation in the onset of acute calcific tendinitis of rotator cuff
Source: BMC Musculoskelet Disord. 2020 Nov 12;21:741. doi: 10.1186/s12891-020-03773-6 (PMC7659130; doi:10.1186/s12891-020-03773-6)
Supplement: Supplementary file 1 — Additional file 1: Supplementary Table 1. Association between the restitution and season of the pain onset of acute calcific tendinitis. [file 12891_2020_3773_MOESM1_ESM.docx]

**Supplementary Table 1. Association between the restitution and season of the pain onset of acute calcific tendinitis**

|  | Winter  n (%) | Spring  n (%) | Summer  n (%) | Autumn  n (%) | P |
| --- | --- | --- | --- | --- | --- |
| Incomplete restitution  (6 months after the pain onset) | 1 (4.0) | 3 (8.1) | 2 (2.8) | 1 (2.5) | 0.667 |

n; number
