# Supplementary material for: Seasonal variation in the onset of acute calcific tendinitis of rotator cuff
Source: BMC Musculoskelet Disord. 2020 Nov 12;21:741. doi: 10.1186/s12891-020-03773-6 (PMC7659130; doi:10.1186/s12891-020-03773-6)
Supplement: Supplementary file 2 — Additional file 2: Supplementary Table 2. Association between the restitution and age of patients in acute calcific tendinitis. [file 12891_2020_3773_MOESM2_ESM.docx]

**Supplementary Table 2. Association between the restitution and age of patients in acute calcific tendinitis**

|  | Restitution (n=148) | Incomplete restitution (n=7) | P |
| --- | --- | --- | --- |
| Age (years) | 64.8 ± 14.1 | 51.9 ± 11.7 | 0.032 |

n; number
